# Stereo electronic principles for selecting fully-protective, chemically-synthesised malaria vaccines

**DOI:** 10.3389/fimmu.2022.926680

**Published:** 2022-10-20

**Authors:** Manuel E. Patarroyo, Adriana Bermudez, Martha P. Alba, Manuel A. Patarroyo, Carlos Suarez, Jorge Aza-Conde, Armando Moreno-Vranich, Magnolia Vanegas

**Affiliations:** ^1^ Grupos: Sintésis Química, Resonancia Magnética Nuclear y Cálculo estructural, Biología Molecular e Inmunología, Fundación Instituto de Inmunología de Colombia (FIDIC), Bogotá, Colombia; ^2^ Universidad Santo Tomas, Bogotá, Colombia; ^3^ Facultad de Ciencias Agropecuarias, Universidad de Ciencias Aplicadas y Ambientales (U.D.C.A), Bogotá, Colombia

**Keywords:** malaria, vaccine, MHC-II-peptide-TCR, IMPIPS, stereo-electronic-relevance

## Abstract

Major histocompatibility class II molecule-peptide-T-cell receptor (MHCII-p-TCR) complex-mediated antigen presentation for a minimal subunit-based, multi-epitope, multistage, chemically-synthesised antimalarial vaccine is essential for inducing an appropriate immune response. Deep understanding of this MHCII-p-TCR complex’s stereo-electronic characteristics is fundamental for vaccine development. This review encapsulates the main principles for achieving such epitopes’ perfect fit into MHC-II human (HLADRβ̞1*) or *Aotus* (Aona DR) molecules. The enormous relevance of several amino acids’ physico-chemical characteristics is analysed in-depth, as is data regarding a 26.5 ± 2.5Å distance between the farthest atoms fitting into HLA-DRβ1* structures’ Pockets 1 to 9, the role of polyproline II-like (PPII_L_) structures having their O and N backbone atoms orientated for establishing H-bonds with specific HLA-DRβ1*-peptide binding region (PBR) residues. The importance of residues having specific charge and orientation towards the TCR for inducing appropriate immune activation, amino acids’ role and that of structures interfering with PPII_L_ formation and other principles are demonstrated which have to be taken into account when designing immune, protection-inducing peptide structures (IMPIPS) against diseases scourging humankind, malaria being one of them.

## Introduction

No vaccines are yet publicly available against HIV, tuberculosis and malaria after almost 50 years of intensive work, billions of dollars invested (1), millions of animal lives sacrificed and hundreds of humans trials involving thousands of people in developing biologically-derived vaccines (2). A new approach is needed; chemistry, physics and mathematics could provide such alternative.

In-depth knowledge acquired throughout these years regarding protein chemistry, structural biology, quantum chemistry, protein and carbohydrate synthesis, along with these molecules’ atomic interactions with immune system molecules and the genetic rules determining appropriate immune responses make this challenge feasible.

This review deals with advances and knowledge acquired by our Institute during the last 40 years pursuing the idea that chemically synthesised vaccines are feasible and the presentation of principles and rules identified along this time in the search for a logical and rational methodology for vaccine development against infectious diseases scourging humankind, malaria being one of them. Our recent research has confirmed that **c**onserved high activity binding peptides (cHABPs) derived from malarial proteins involved in relevant parasite functions during invasion do not induce immune responses during infection (therefore being non-antigenic); consequently, they must be properly modified (following the previously described rules) to make them highly immunogenic and protection-inducing (modified high activity binding peptides - mHABPs) (3–5). The criteria for selecting the proteins mentioned in this manuscript were based on previous studies related to evaluating their immune response, their mHABP structure (as determined by potent ^1^H-NMR), molecular modelling of the fit into HLA-DRβ1* molecules and bioinformatic tools. Those mHABPs having the best results emphasising their stereo-electronic principles were selected.

This review thus provides a brief description of a large panel of peptides’ stereo-electronic, physico-chemical and structural characteristics as identified in malaria parasite proteins and their interplay with human host immune system molecules regarding our goal for developing fully-protective, chemically-synthesised anti-malarial vaccines.

## The malarial parasite’s development cycle

Malaria continues being a serious public health problem worldwide having induced 241 million cases and 627,000 malaria-related deaths in 2020 (World Malaria Report 2021) (6), *Plasmodium falciparum* being responsible for the greatest mortality. The forgoing suggests that a prophylactic method is urgently needed, i.e. a completely effective vaccine.

**Figure 1:**
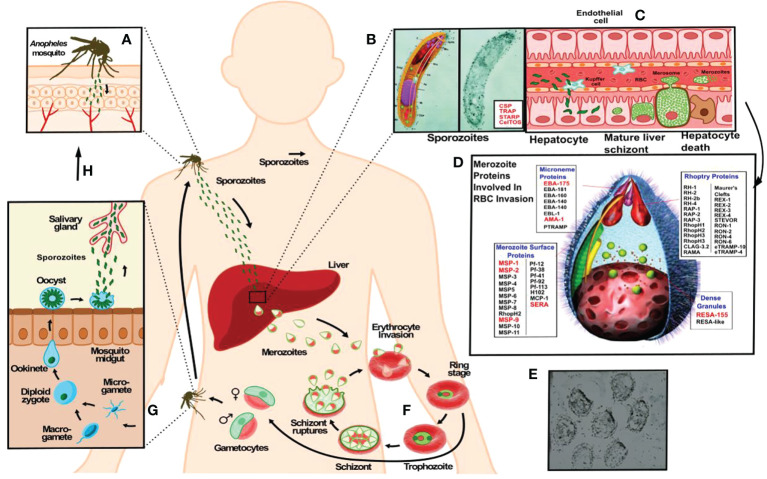
The *P. falciparum* malarial parasite has two infective forms regarding its human host. A female *Anopheles* mosquito injects **(A)** a larvae-like structure or sporozoite (Spz) **(B)** during her blood meal (<1,000 Spz/bite). These migrate to the liver to infect hepatic cells where each reproduces ~30,000 times during a single week **(C)**, changing their morphology and molecules to yield the second infective form: the merozoite (Mrz). This is a pear-like structure **(D, E)** which invades red blood cells (RBCs) where it reproduces ~32-50 times each 48 hours **(F)** to infect an equal number of RBC, thereby producing the clinical symptoms of this disease: episodes of very high fever followed by chills, headache, nausea, vomiting, malaise, abdominal and muscle pain, anaemia, cerebral and renal malaria. The latter leads to the death of sick patients when not properly and/or opportunely treated. Another stage consists of gametes **(G)** (male and female) infecting a new mosquito **(H)**, thereby continuing the reproductive cycle to infect other human beings **(A)**.

However, developing an anti-malarial vaccine has been extremely difficult due to the parasite deploying a large number of proteins having broad genetic variability (7, 8) expressed during its development stages inside a human host, also deploying wide-ranging genetic polymorphism concerning human immune system molecules. There is also the complexity of host-parasite interactions enabling the parasite to evade a host’s immune response. **Figure 1** briefly explains the *P. falciparum* malaria parasite’s cycle.

## Historical background regarding synthetic anti-malarial vaccine development and adopting a new approach

Our original work began by 1984 when we developed the first chemically-synthesised vaccine (9) which was tested in *Aotus* monkeys using a mixture of short peptides derived from 3 merozoite proteins. They provided sterile protective immunity in some vaccinated monkeys, demonstrating that chemically synthesised vaccines were feasible. These peptides plus one sporozoite sequence inserted twice were further synthesised as a unique 45-mer polymerised (via cysteine added at the N- and C-terminus) polypeptide, named S*Pf*66.

The SPf66 chemically-synthesised vaccine (also called “Colombian synthetic vaccine”) was the first multi-epitope, multistage, chimeric (3 merozoite- and one sporozoite-derived proteins peptides in a single molecule) vaccine, tested in *Aotus* monkeys. It was highly immunogenic, as assessed by immunological methods, i.e. immunofluorescence antibody test (IFA) and Western blot (WB), and induced full protection/immunity in some monkeys. However, it was clearly seen from the beginning that more epitopes were needed for SPf66.

SPf66 component peptides were later seen to be involved in host-cell invasion, clearly suggesting that high activity binding peptides (HABP) derived from some proteins involved in host cell invasion had to be included in S*Pf*66. A highly specific and sensitive radiolabelling methodology was thus developed for recognising 20-mer long, non-overlapping, sequential peptides having high binding activity, covering the complete protein’s amino acids sequence. This approach summarised in a review led to identifying HABPs in 38/54 merozoite and 15 sporozoite proteins previously shown to be involved in RBC and hepatocyte invasion by Florens et al. (10). Some variable HABPs had highly immunogenic aa sequences (vHABP) as a way of escaping host immune response, whilst another group was highly conserved (cHABPs), mostly performing relevant biological functions. It was thus decided to work with cHABPs (11).

Immunising *Aotus* monkeys with cHABP proved very disappointing. None were immunogenic or protection-inducing whereas vHABPs were highly immunogenic but strain-specific. This “gordian knot” was cut when some amino acids were randomly replaced by glycine, thereby inducing immunogenicity and protective immunity in some of them. A large group of mHABPs were found to be highly immunogenic and protection-inducing in a large series of studies including hundreds of mHABPs tested in thousands of *Aotus* monkeys (reviewed in (3). Such in-depth, extensive work found that some residues had to be replaced by others having the same mass and volume but opposite polarity (reviewed in (11).

Developing a logical and rational methodology for vaccine development using potent ^1^H-NMR (600 -500 MHz) determined ~600 cHABP and mHABP 3D structure to determine their structural differences and rules or principles for immunogenicity and/or protection induction. It was found that such modifications concerned the capability of forming an appropriate MHCII-p-TCR complex and related stereo-electronic principles. An in-depth, thorough analysis of some existing rules and new ones are this manuscript’s *raison d’être*.

## Synthetic vaccine development strategy

*P. falciparum* causes the most lethal form of human malaria; it is transmitted by the bite of an infected female *Anopheles* mosquito. The parasite has a genome encoding ~5,600 proteins performing different biological functions (12), ~30 of them used by sporozoites (Spz) to migrate, invade endothelial, Kupffer and hepatic cells (10) (**Figure 1C**) and ~50 more used by merozoites (Mrz) (**Figures 1D, E**) to invade RBCs (13) (**Figure 1F**) in a receptor-ligand-type interaction. Such highly sensitive, specific and robust methodology recognised ~200 peptides interacting with receptor cells (11, 14) for identifying minimal protein subunits as vaccine targets.

**Figure 2:**
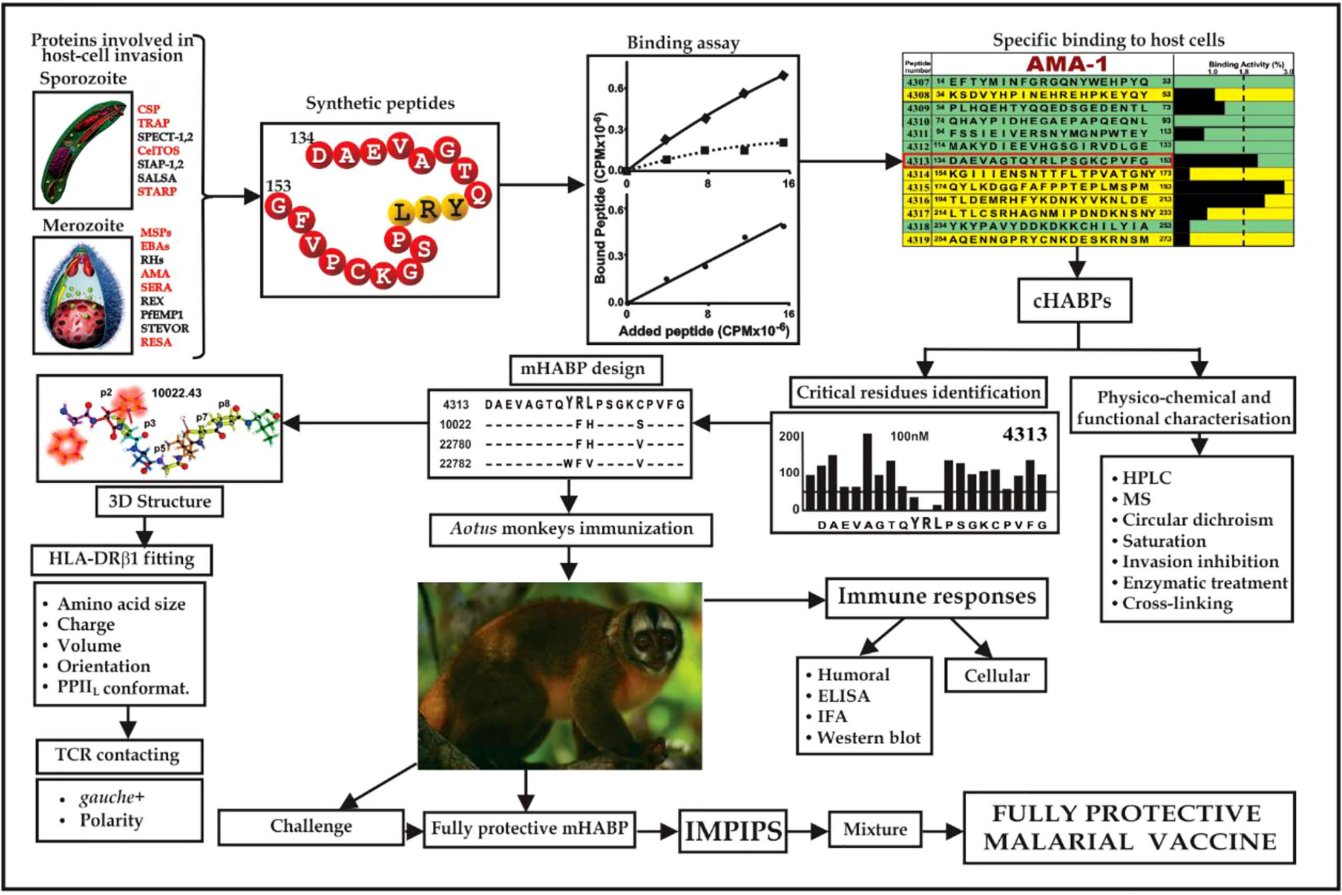
Flowchart for fully-protective malaria vaccine development, based on elegant work by other groups and in-depth reviews (4, 11, 14). Spz- and Mrz-derived proteins’ aa sequences relevant in *P. falciparum* invasion; 15-20-mer cHABP synthetic peptide evolution for developing a protective, minimal subunit-based, multistage, multiepitope anti-malarial vaccine, following established stereo-electron principles (3, 5, 15) (Taking from Ref. 15).

**Figure 3:**
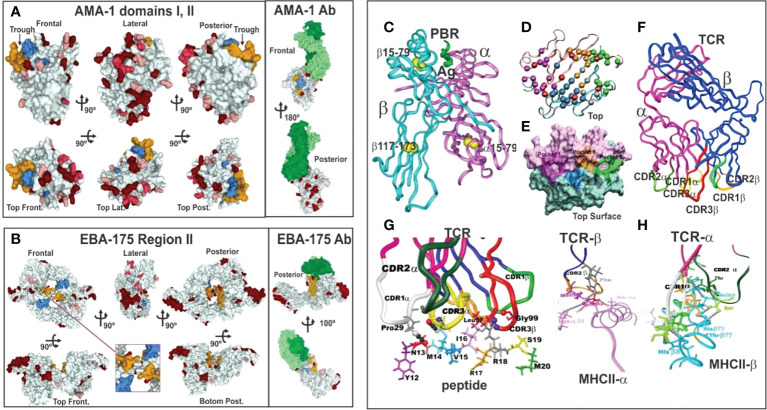
**(A, B)** 3D representation and polymorphism analysis of AMA-1 domain I and EBA-175 RII with mAb complex.Taking from Ref. 32. **(A)** cHABPs 4313 (blue) and 4325 (dark yellow) are far away from highly polymorphic regions in the AMA-1 recombinant fragment to which 1F9 mAb bind (lesser to greater polymorphism shown by pink, red to burgundy). **(B)** EBA-175 RII dimer showing hand-shake structure cHABPs 1779 (blue) and 1783 (dark yellow) in the box; alongside, mAb R218 bound far away from these cHABPs in the F1 domain. **(C)** Front view of class II (HLA-DRβ1*0101) molecule 3D structure (PDB code: 1DLH) (16), showing the α-chain (light pink) and β-chain (clear blue ribbon). **(D)** Top-view, ribbon representation of HLA-DRβ1*0401, showing α- and β-chain aa forming Pockets 1 (fuchsia), 4 (dark blue), 6 (light brown) and 9 (green). **(E)** Front view, Connolly representation of HLA-DRβ1*0101 3D structure with α- and β-chains forming the PBR where mHABP aa sidechains will be accommodated (according to established colour code). **(F)** TCR-α (dark pink) and β-chain (dark blue) with CDR1α (white), CDR2α (dark green), **(G)** CDR3α (yellow), CDR1β (light green), CDR2β (orange) and CDR3β (red) above peptide 24112 showing interaction *via* H-bonds (grey balls) with HA-1.7TCR, modified according to Vβ12 clone 3 sequence from protected *Aotus* monkeys. **(H)** TCR interactions with MHC α- and β-residues to stabilise complex binding (**C, D, F–H** taking from Ref. 3).

This work highlighted that functionally-relevant, cHABPs from most of the parasite’s important proteins involved in host cell invasion must be recognised (**Figure 2**). However, cHABPs are immunologically silent (due to their 3D structure) for escaping host immune response (**Figure 3**); they must thus be properly modified to become highly immunogenic and protection-inducing (i.e. mHABPs) (**Figure 2**), indicating their derivative nature (3–5, 15).

Such modified, minimal subunit-based peptides must fulfil a set of physico-chemical, electronic and topological characteristics (3–5, 17, 18) for an appropriate fit into MHCII-p-TCR complex (the first and critical step in immune activation: antigen presentation) to induce an appropriate immune response (**Figure 3**).

This review thus covers peptides’ immunological behaviour based on their amino acids’ (aa) physico-chemical characteristics for fitting into the trimer MHC-II-p-TCR complex. Stereo-electronic and topochemical parameters are analysed, i.e. the **distance** between the furthest atoms of a peptide’s residues fitting into MHCII groove (peptide binding region (PBR)) pockets (17, 18), their charge, volume, location and other physico-chemical parameters, i.e. mHABPs must be or contain left-handed polyproline II helix (PPII_L_)-like structures (19–21) to properly fit into this PBR segment (17, 22). The sidechain orientation of aa fitting into HLA-DRβ* or Aona DR PBR (human or *Aotus* monkeys’ class II molecules associated with antibody (Ab) production) and sidechain *gauche+* orientation for aa in peptide positions 3 and 7 (p3 and p7) are needed for appropriate interaction with the TCR (23). Electrostatic forces in the peptide region binding to the MHCII groove, H-bonds and π-CH (24, 25), π-cation (26, 27), π-SH (28), π-π (29), n—>π* interactions (30, 31), needed for stabilising other molecular structures must be replaced due to their tendency to disturb PPII_L_ formation. The results shown here and the forgoing stereochemical and topological parameters analysed at FIDIC are based on 3D structure determination by powerful ^1^H-NMR (500-600 MHz) spectroscopy of ~600 functionally, immunologically and immuno-genetically studied peptides.

## cHABPs are immunologically silent

cHABPs mediating important biological functions for parasite invasion and survival are immunologically silent, i.e. cannot induce an immune response. They are not immunogenic, as thoroughly shown in the ideal experimental model (*Aotus* monkeys); they are not antigenic following *P. falciparum* infection as shown by countless epidemiological and immunological studies.

Such silence has been elegantly demonstrated by X-ray crystallography. A panel of molecules complexed with monoclonal antibodies (mAb) (**Figure 3**) (3, 32) are shown as hypervariable regions (highlighted in red, the darker the more variable) and yellow and blue indicates AMA-1 aa in cHABP 4313 and 4325 in protein surface structure. The interaction of some hypervariable regions with murine mAb (1F9) is shown (H-chain in dark green and L-chain clear) far away from cHABPs 4313 and 4325 (**Figure 3A**). EBA-175 binding to RBC glycophorin A glycans 2, 5 and 6, cHABPs 1779 (**Figure 3B** pale blue, not included in this study) and 1783 (dark yellow), located in the recombinant fragment containing RII and F1 segments, clearly shows this molecule’s tremendous polymorphism (many dark red areas). mAb R218 (green) reactivity targeted a region far away from cHABPs 1779 and 1783 located within molecules.

**Figure 4:**
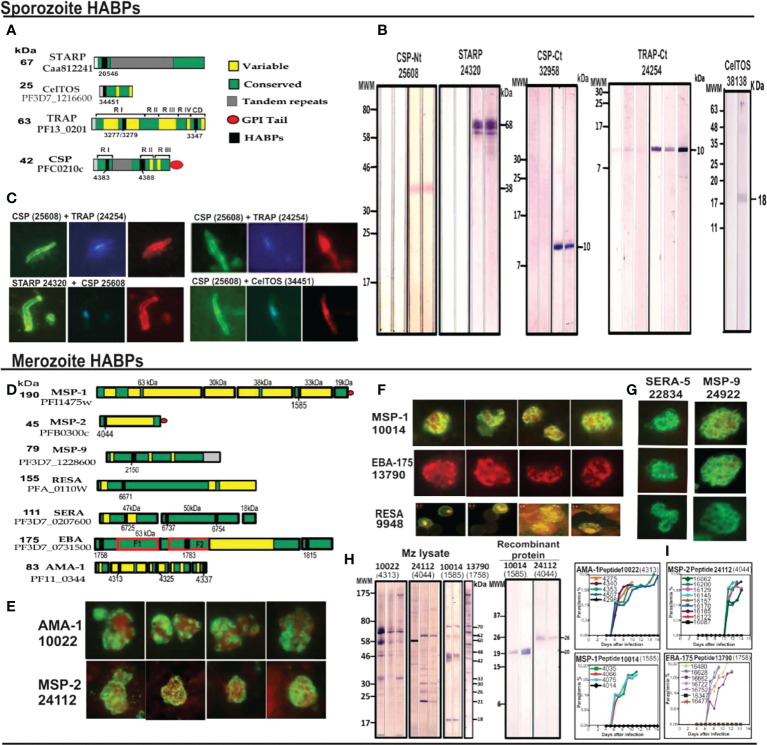
cHABP location in proteins and their mHABPs’ immunogenicity. **(A)** Diagrammatic representation: bar length represents their appropriate molecular weight (MW). Each cHABP location is indicated by the institute’s number code shown below. **(B)** Western blot (WB) analysis of recombinant fragments produced in our Institute covering different protein regions (the STARP recombinant fragment was kindly provided by Prof. Pierre Druille, Pasteur Institute, Paris, France). **(C)** Immunofluorescence patterns regarding sera from immunised *Aotus* monkeys, showing reactivity with CSP-1 and STARP (green fluorescence) on Spz membrane and TRAP and CelTOS intracytoplasmic location (red fluorescence). **(D)** Diagrammatic location of *P. falciparum* proteins involved in RBC invasion. **(E–G)** IFA patterns for *P. falciparum* Mrz, showing reactivity with membrane proteins MSP1, MSP2, MSP9, rhoptry proteins EBA-175, AMA-1, SERA and infected erythrocyte (iE) membrane (RESA). **(H)** Immunofluorescence patterns regarding sera from immunised *Aotus* monkeys, showing reactivity with AMA-1, MSP-1, MSP-2 and EBA-15 (green fluorescence) on Mrz membrane intracytoplasmic location (red fluorescence). **(I)** The course of parasitemia in *Aotus* monkeys immunized with fully protection inducing peptides. Adapted from Ref. 16, 17.

These and many more examples (32) represent clear structural evidence of cHABPs’ immunological silence, confirmed by a different method, and that the immune response mainly targeted variable aa sequences. The cHABPs, located in the protein region shown in green (**Figures 4A, D**).

Protection-inducing mHABPs have been called IMPIPS for several years now (5) (number in bold for mHABPs and numbers in ordinary text or in parenthesis for cHABPs). These peptides’ immunogenicity has been thoroughly reported by immunofluorescence antibody assays (IFA) and their reactivity with Mrz lysates (**Figures 4E–G**) or recombinant Spz (**Figure 4B**) or Mrz (**Figure 4H**) protein fragments by WB (**Figures 4B, H**). IFA detected proteins exact locations in the Spz (**Figure 4C**) or Mrz (**Figures 4E–G**) organelles from which the proteins’ aa sequences were obtained and the peptides derived. WB showed strong reactivity against native (as with Spz or Mrz lysates) or recombinant proteins fragments (3–5, 11, 14, 15). These Mrz mHABPs induced sterile protective immunity during experimental challenge in some monkeys (**Figure 4I**).

High Ab titres indicated intense immune response; however, immunoglobulin class and subclass so induced must be analysed due to the genetic control exerted on them *via* genetic (Gm+) markers, mainly in IgG 1,2,3,4 subclasses. Ab affinity must also be taken into account when correlating with protection.

## mHABP selection

### mHABP analysis

Forty-six peptides were analysed based on their aa sequences and potent ^1^H-NMR (500- 600 MHz) spectroscopy that determined their 3D structure; 17 of them were highly immunogenic and protection-inducing. Ten more peptides STARP-, CSP-1-, TRAP- and CelTOS-derived proteins were also included (**Figures 8**–**12**) as being relevant during the first step of *P. falciparum* Spz invasion of liver cells and motility for cell traversal, seven being highly immunogenic, and 9 native cHABPs to compare their physico-chemical characteristics.

### Protection-inducing immune response capability

The 17 Mrz mHABPs were derived from MSP-1 11860 (1585), MSP-2 10014 (1585), 10008 (4044), 24112 (4044), MSP-9 or ABRA 24922.37 (2150), RESA 13492.36 (6671), 9948.4 (6671), SERA-5 22834.42 (6737), 23426.2 (6754), 22830.25 (6725), EBA-175 22814 (1783), 24292.12 (1815), 13790.46 (1758) and AMA-1 20032.35 (4325) 20034.32 (4325), 22780 (4313), 10022.43 (4313) (**Figures 8**–**11**). They induced protective immunity determined by challenge following the last immunisation (**Figure 4I**). Protection was defined as the total absence of parasites in challenged monkeys’ blood during the 15-day follow-up of challenge with 100,000 infectious erythrocytes freshly obtained from another naïve, infected monkey (3, 15). Monkeys immunised with the immunogenic but non-protection-inducing mHABPs and their cHABPs had parasites in blood from day 4 onwards (reaching ≥2.5% on days 8 or 9).

## Host immune system molecules

### MHC Class II or HLA-DRβ* antigen-presenting molecules

Elegant immunogenetic, biochemical, immunological and X-ray crystallography studies have shown that MHCII molecules are heterodimers encoded in humans by a genetic region located in chromosome 6 short arm (HLA-D), having different sub-regions named HLA-DP, DQ and DR. The latter has a relatively monomorphic α-chain (**Figures 3C, D**, pink ribbon) and a highly variable β-chain (**Figures 3C, D** pale blue ribbon) encoded by nine genes (five being pseudogenes which are non-transcribed, non-expressed); expressed ones are named HLA-DRβ5*, β4*, β*3 and β1*. Highly polymorphic HLA-DRβ1* encodes 16 allele families (HLA-DRβ1*01-16) having ~2,500 alleles based on their aa sequences, with different genetic frequencies in ethnic groups worldwide. The α- and β-chains create a narrow, long, deep groove named the peptide binding region (PBR) (**Figure 3C**) determining antigen specificity according to their aa sequences (i.e. Pockets 1, 4, 6 and 9) (**Figures 3D, E**) (16).

Please note that from here on an established colour code for figures produced by our group has been used: light pink represents HLA-DRβ1* α-chain and pale blue the β-chain (**Figures 3C–E**). Fuchsia represents residues forming Pocket 1, deep blue Pocket 4, orange Pocket 6 and green Pocket 9 (**Figures 3D, E**).

**Figure 5:**
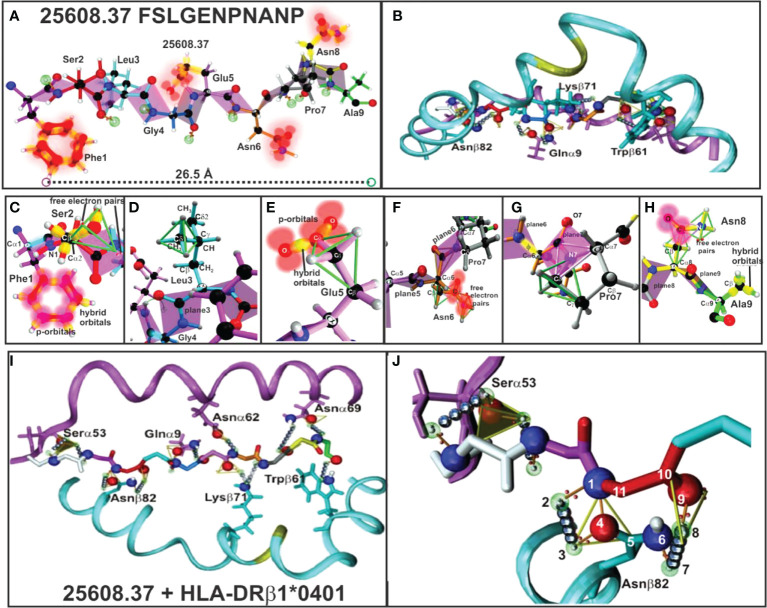
**(A, B)** 25608.37 mHABP plane steric electron characteristics: peptide-bond formation (rose), σ and π bond (stick) between each atom, p-orbitals perpendicular to them (blurred red balloons). Phe1 shows π resonance (red bonds between p-orbitals). **(C)** Ser2: tetrahedron having 2 free electron pairs, showing only the σ-bond (yellow). **(D)** Leu3: showing the tetrahedron framing the Cσ1 sidechain; only t plane 3 is shown for Gly4. **(E)** Glu5: tetrahedron framing Cγ and trigon in resonance between two O with Cγ from the sidechain. **(F)** Asn6: horizontally orientated towards P6, showing the tetrahedron, a trigon and electron charge of 2 free orbitals (blurred red). **(G)** Pro7: (grey) 2 trigons (green). **(H)** Asn8: pointing upwards towards the TCR with p-orbitals and non-bonding free electron pair and Ala 9 (green) with hybrid orbitals (green tetrahedron). **(I)** H-bonds between HLA-DRβ1*0401 aa sidechains and 25608.37 backbone atoms (silver balloons). The 11 H-bonds establishing fork-like ring structure between Asnβ82 and 25608.37 Ser2. **(J)** Side view of H-bonds (silver balloons) between HLA-DRβ1*0401 Nβ82, Qα9, Kβ71 and Wβ71 aa sidechains with 25608.37 backbone atoms. (Adapted from Ref. 5).

**Figure 6:**
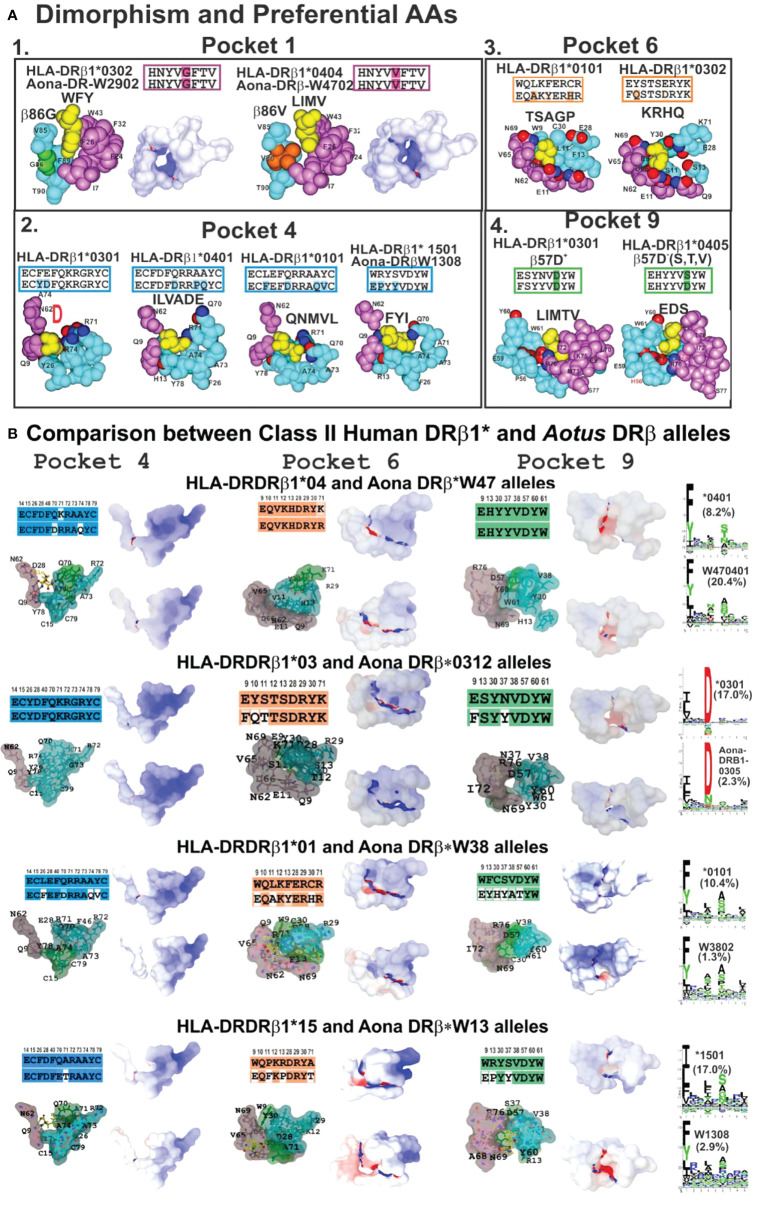
**(A)** P1 dimorphism and preferred aa for P4, P6 and P9, (A1, 2, 3, 4) showing the space required to fit a peptide residue into each pocket. **(B)** Comparing class II human DRβ1* and Aona DRβ allele structures in P4, P6 and P9 with their aa sequences (according to established colour code) and aa differences between these 2 species (uncoloured). Surfaces having electrostatic differences: electronegative (blue) and electropositive surfaces (red). Left-hand side: logos for each HLA-DRβ1* and *Aotus* DR allele; their frequency shown in parenthesis. Adapted from Ref. (33).

All peptides’ stereo-electronic and topochemical parameters influencing suitable peptide fit into the MHCII-PBR were analysed. This included atomic analysis of the 9 aa fitting into the MHCII PBR, a peptide’s correct sidechain occupation [by volume (**Figure 6A**)], charge (**Figure 6A3**) and orientation (**Figures 5B–H**) in Pocket 1 to 9 determined by non-covalent interactions (H-bonds) where peptide stabilisation in the PBR (16, 34, 35) (**Figure 5I**) guaranteed high affinity binding and epitope stability.

HLA-DR β1* α-chain Serα53, Asn α62, Asnα69, Gluα9 and Asnβ82, Lysβ71 and Trpβ61 (**Figures 5I, J**) located in the PBR led to peptide stabilisation establishing H-bonds with the aa backbone. Such stable structure was established by aa forming trigons with the –p1(S) residue in Serα53 and a p2(Ser) conforming an 11-atom, fork-like ring structure (N,O and free electron) forming trigons (**Figure 5J**) (36), along with αGlu9 with Pocket 4 (Gly) backbone, Asnα62 with Pocket 6 (Asn), Lysβ71 with p7 (Pro); Asnα69 with p8 (Asn) and Trpβ61 with Pocket 9 (Ala) backbone.

The other IMPIPS-related aa fitting into the PBR were solvent-exposed and upwardly-orientated to interact with the TCR. Their positions in a peptide are designated by the letter p, i.e. p2 red, p3 pale blue, p5 pink, p7 grey and p8 yellow.

### TCR molecule characteristics

Other mHABP residue sidechains (numbered according to their positions, preceded by a p) had to perfectly dock with TCR-contacting residues (TCRCR) to form the appropriate MHCII-p-TCR complex (or immunological synapse) during antigen presentation (37) for correctly activating the immune system.

Classical X-ray crystallography studies have demonstrated that the TCR is a heterodimer molecule (α-chain dark pink and β-chain dark blue), having **c**onserved (Cα and Cβ) (**Figure 3F**) and **v**ariable (Vα,Vβ) regions with 6 TCR loops located at the variable β-strand loops of α- and β-chains (37), i.e. complementary determining regions (CDR1, 2, 3) (**Figures 3F–H**). It has been shown that the TCR adopts a diagonal orientation in which Vα (white) lies on top of MHCII β-helices and a peptide’s N-terminus and Vβ (orange) contacts MHCII α-helices and the C-terminal portion (**Figures 3G, H**). Relatively conserved CDR1α (white), 2α (deep green) and CDR1β (light green) and 2β (orange) loops generated by germ-line-encoded residues lay over MHCII protein helices (**Figure 3G**); hypervariable CDR3α (yellow) loops produced by genetic rearrangement (i.e. antigen-selected variability) tended to make contact with peptide 24112 residues M14 whilst hypervariable CDR3β (red) contacted R18 (**Figure 3G**) (38, 39). This provided such interaction’s strong specificity and tremendous variability.

The difference in polarity of TCR contact residues p2 and p3, p5, p7 and p8 could also be associated with mHABPs’ protection-inducing immunity as the aa in p2, p5 and p8 were charged, had p orbitals or non-binding electron pairs in their molecular structure and most aa in p3 and p7 were aliphatic, or non-polar.

Seventeen immunogenic, protection-inducing (IMPIPS), 15 immunogenic non-protection-inducing, 5 non-immunogenic mHABPs and the 9 native cHABPs from which they were derived are described and analysed to explain their behaviour.

### Determining HLA-DRβ1* binding motifs

The NetMHCIIpan-3.1 platform available in 2013, having (40) high specificity (~80%) and high sensitivity (~90%), was used for predicting (80%) HLA-DRβ1* binding peptide cores (previously determined by X-ray crystallography). Results agreed with some of our experimentally-determined values for IMPIPS binding to purified HLA-DRβ1* molecules (3–5), thereby corroborating our finding that mHABPs must be specifically modified to fit into specific HLA-DRβ1* molecules to induce the appropriate immune response.

NetMHCIIpan-3.1 DNA sequence determination of around 900 *Aotus* monkeys’ MHCII (Aona-DR) genome (41–43) was complemented by predicting Aona-DR allele similarity (S) or identity (I) with HLA-DRβ1* alleles to determine which IMPIPS could be used in humans without further modification the **Figure 6** shows the comparison of electrostatic potential between HLA-DRβ1* and Aona-DRβ Pockets 4, 6 and 9 with their corresponding aa differences, their surface landscapes and the predicted aa fitting into alleles. This approach dramatically reduced the amount of expensive, risky, difficult to perform and analyse clinical human trials, involving many people, some lasting years. NetMHCIIpan4.1 was used from then on (i.e. higher sensitivity and specificity) (40).

## Protein and dihedral angle secondary structure

Almost 60 years ago (1963), the crystallographer G.N. Ramachandran et al., using the little information available by then, impressively depicted planar amide bond geometry in a simple figure (Ramachandran Plot) having two dihedral angles: φ on the *X* axis and ψ on the *Y* axis (44), clustering a protein’s major folds in only a few sections of the map. They used very simple stereochemical predictions of protein backbone covalent bonds to predict that the α-helix and extended β-sheet-like structure should show that the carbonyl C=O double bond cannot rotate. The carbonyl C atom and the amide N atom have only two possible ω angle geometries due to the C-N bond’s partial double bond characteristic: *cis* where the ω angle has 0° value or *trans* where ω is 180°. N-Cα bond rotation can be determined by the C*_i-1_*-N-Cα-C dihedral angle (φ) while the Cα-C bond can be measured by the N-Cα-C-N*_i+1_* angle (ψ). An inter-atom clash is unacceptable since the atoms cannot penetrate each other; a collision thus occurs when the distance between 2 atoms is smaller than the sum of their van der Waals radii. These were the predictions by Ramachandran et-al that lead to the very useful and quoted Ramachandran plot (44).

Elegant work by protein chemists and structural biologists has enlarged the list of protein and peptide secondary structures based on traditional interactions stabilising protein structures: Lennard-Jones potentials (steric clashes) and H-bonds (45).

**Figure 7:**
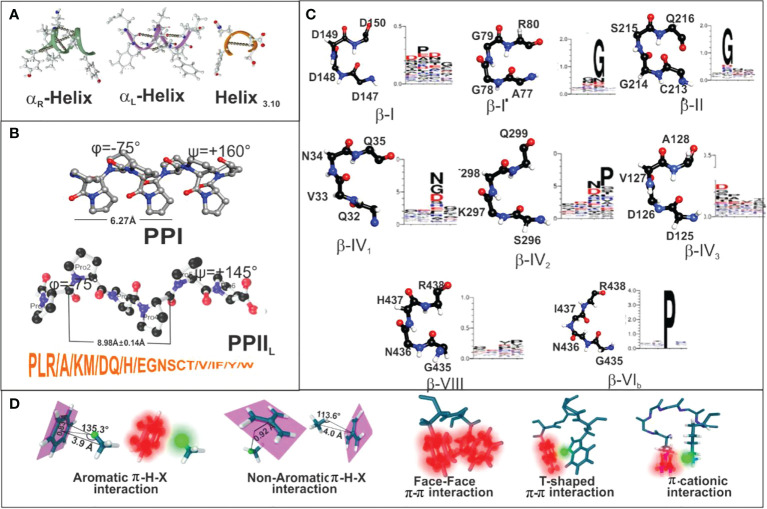
**(A–C)** Secondary structure features for helix_3.10_, αR helix, αL helix, beta-strand, PPI and PPII conformations, along with the most recognised β-turns mentioned here. **(D)** Summary of peptide π-interaction system (blurred red) and interactive hydrogen (blurred green). Taking from Ref. (49).

The International Union of Pure and Applied Chemistry (IUPAC) and the International Union of Biochemistry and Molecular Biology  (46) have stated that peptides and proteins ideally have an 3_10_-helix having eight residue segments and φ=-60.0°, ψ=-30.0°, ω=+180° torsion angles. The 3.6 residues in α-right-handed (α_R_) helixes typically adopt identical or quite similar backbone φ, ψ, ω dihedral angles (-57°, -47°, +180°) (47, 48) while α-left-helix (α_L_) have (+60°, +52°, +180°) angles (**Figure 7A**). β-strands have five ten-residue segments with -119° φ, +113° ψ and +180° ω for parallel-strands and -139°, +135° and +180° torsion angles for antiparallel-strands (**Figure 7A**) (50). PPII_L_ helixes have φ=-75° ± 25, ψ= +145° ± 25, ω=+180° torsion angles (**Figure 7B**) and symmetrical sPPII helixes have -145°, +80.9, +180° and φ=-83°, ψ=+158 and ω=0° for PPI helixes (**Figure 7B**) (20, 51). β-turns (the most commonly recognised protein structure), consisting mainly of four aa, assume many different types, (I to VIII being most representative), distinguished by *i+1* and *i+2* residues’ φ, ψ angles where ±30° deviation from canonical values is allowed for 3 of these angles and ±45° for the fourth (**Figure 7C**) (52, 53).

Peptides’ partial double-bond nature in proteins determines *cis* (ω=0°) and *trans* (ω=180°), depending on dihedral angle ω value [C_α_(1)-C(1)-N(1′)′-Cα(1′)] (54). These conformations are very relevant since PPII_L_ are always in *trans* and PPI in *cis* (**Figure 7B**). Such stereo-electronic characteristics determining protein and peptide secondary structure determine the logical and rational methodology for vaccine development.

## PPII_L_ structures as vaccine components

Polyproline type-II, left-handed (PPII_L_) structures, together with α-helixes, β-sheets and β-turns, are among the most abundant secondary structures in proteins and peptides, playing very relevant roles in biological processes (55). Elegant work by Jardtzky et al. (22), based on X-ray crystallography data from MHC II-peptide complexes and confirmed by others in the 1990s, clearly demonstrated that antigenic (16, 56, 57) and immunogenic peptides (58–60) have PPII_L_ structure where they interact with the MHC II region, i.e. PBR (**Figures 5A, I**).

We began a search around 2001 for ways to make IMPIPS PPII_L_-propensity formers. Our recent structural studies in *Aotus* monkeys have shown that IMPIPS have/contain PPII_L_-like structures regarding fragment binding to the PBR (17, 18, 61). This confirmed that epitopes should have or contain PPII_L_-like for immunogenicity and protection-inducing immunity during highly stringent intravenous challenge with the very virulent *P. falciparum* FVO, *Aotus*-adapted strain.

Antigen presentation must thereby involve a deep understanding of PPII_L_ characteristics and the inherent stereo-electronic characteristics regarding logical and rational vaccine development methodology.

## Relevant IMPIPS amino acid stereo-electronic signature

### Amino acid predilections and inter-atom distances as determinant factors

A large set of physico-chemical rules (3, 4, 11, 17, 18, 23, 61) had to be followed for IMPIPS design regarding appropriate antigen presentation and immune activation involving the design of IMPIPS formed by or containing PPII_L_-propensity former structures for properly fitting into the HLA-DRβ1*PBR.

PPII_L_ are distinct secondary structure elements regarding their sequence and structure, having left-handed geometry, as opposed to other helical structures like α-helixes having right-handed geometry. PPII_L_ are heavily solvent-hydrated due to their backbone’s high solvent accessibility (62).

Ideal PPII_L_ helixes are contained in 3 residues per turn; structures should be 3-13 aa long (63), have 9.1 Å (8.98 ± 0.14 Å) pitch distance per turn (62, 64, 65), all amide bonds in trans (ω=180°), sidechains nearly perpendicular to the peptide’s backbone and an average φ=-75°± 25 and ψ=+145°± 25 backbone dihedral angles (**Figure 7B**) (17, 18). Roughly 70% of PPII_L_ structures have 1 or 2 Pro and ~25% of them none at all (64, 66).

The aforementioned physico-chemical rules thus led to ideal IMPIPS design involving P>> L> R> A> K>M/D>Q/H, E> G/N/S/T/V/I/F/Y/W aa propensity for PPII_L_ formation (20) (**Figure 7B**). Such propensity was based on extensive PPII_L_ 3D structural analysis of proteins in the Protein Data Bank (PDB) (51, 66, 67) and exhaustive, straight-forwarded host-guest studies with synthetic peptides to determine PPII_L-_forming propensity (68–71) (**Figure 7B**) and PPII_L_-propensity studies (19, 72). Although PPII_L_ lack intra-chain H-bonds, some residues like S, T, Q, N stabilise secondary structure by non-local H-bond formation with the backbone (short chain-backbone or SC-BB interaction), disturbing PPII_L_ formation (51, 67, 73).

**Figure 8:**
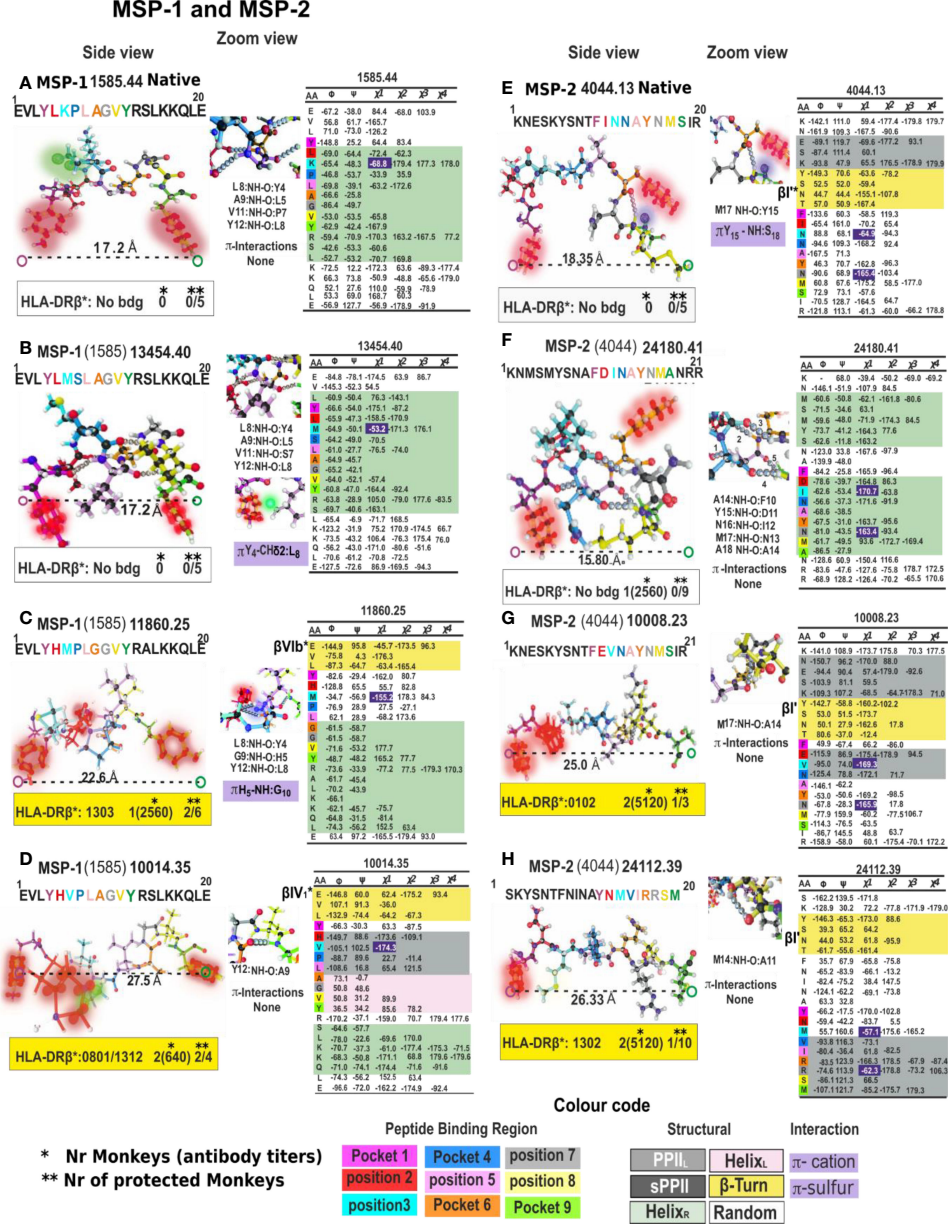
Right-hand panel: Dihedral angles table showing powerful 1H-NMR cHABPs and mHABPs conformers angle determinations, with representative torsion angles (ϕ, ψ, χ1, χ2, χ3 and χ4) in native; non-immunogenic, non-protection-inducing; immunogenic, non-protection-inducing mHABPs and immunogenic, protection-inducing mHABPs or IMPIPS. In dihedral angles table, PPIIL region (grey), sPPII region (dark grey), β-turns (yellow), right-handed alpha helix (αR green), left-handed alpha helix (αL (pink) and random coil structure (uncoloured). χ1 angles for P3 and P7 highlighting gauche+ rotamer orientation (purple) (22). Left-hand panel: Side view of cHABP and mHABP structures (1H-NMR) in the binding region to HLA-DRβ1*: aa sequence above (binding residues coloured according to colour code shown below: Pocket 1 (fuchsia), Pocket 4 (dark blue), Pocket 6 (orange), Pocket 9 (green), position 2 (red), position 3 (light blue), position 5 (pink), position 7 (grey), position 8 (yellow)). Distances between the most distant residues fitting into HLA-DRβ1*PBR Pockets 1-9 (dotted lines are measurements in Angstroms (Å)). Boxed IFA titres and the amount of monkeys protected after challenge (yellow). (*) Nr Monkeys (antibody titers) and (**) Nr of protected Monkeys. Zoom shows intramolecular H-bonds and π interactions (31) between the atoms described below (dotted light grey balls). For this figure, MSP-1- and MSP-2-derived cHABPs and mHABPs.

**Figure 9:**
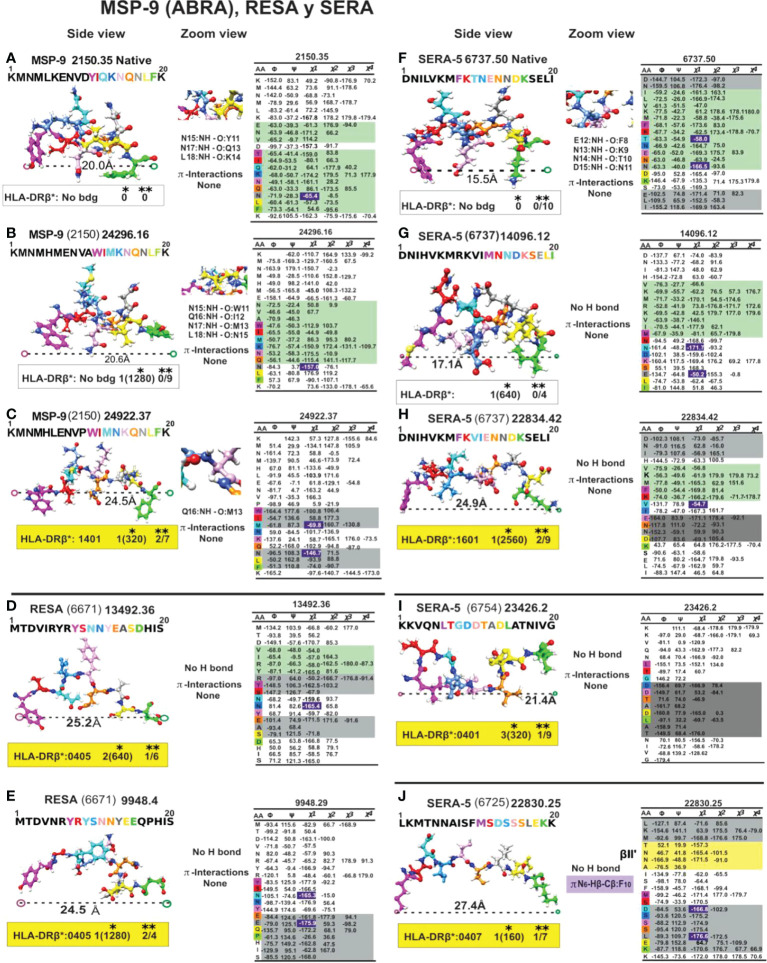
Colour code for peptide binding region, structural and interaction as in **Figure 8**. (*) Nr Monkeys (antibody titers) and (**) Nr of protected monkeys. MSP-9-, RESA- and SERA-derived cHABP and mHABPs. Legend and conventions as in **Figure 8**.

**Figure 10:**
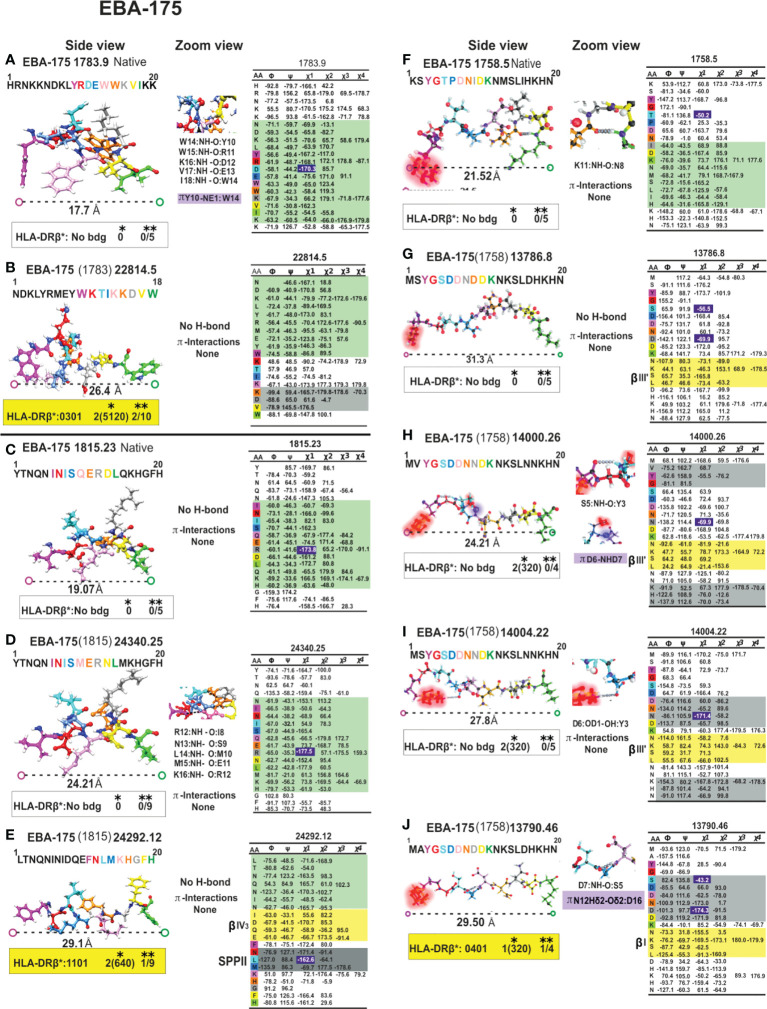
Colour code for peptide binding region, structural and interaction as in **Figure 8**. (*) Nr Monkeys (antibody titers) and (**) Nr of protected monkeys. EBA-175-derived cHABPs and mHABPs. Legend and conventions as in **Figure 8**.

**Figure 11:**
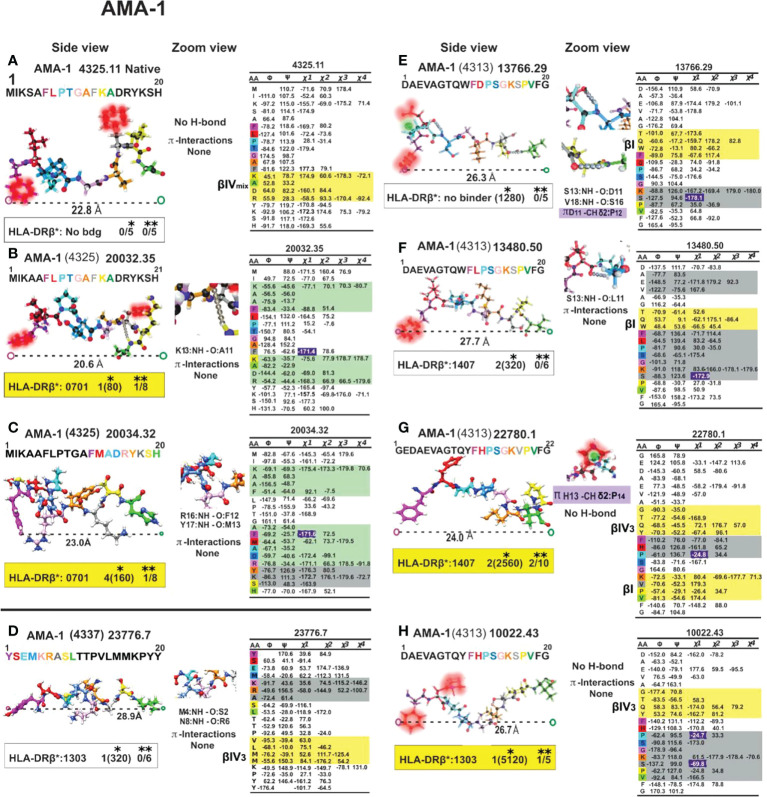
Colour code for peptide binding region, structural and interaction as in **Figure 8**. (*) Nr Monkeys (antibody titers) and (**) Nr of protected monkeys. AMA-1-derived cHABPs and mHABPs. Legend and conventions as in **Figure 8**.

**Figure 12:**
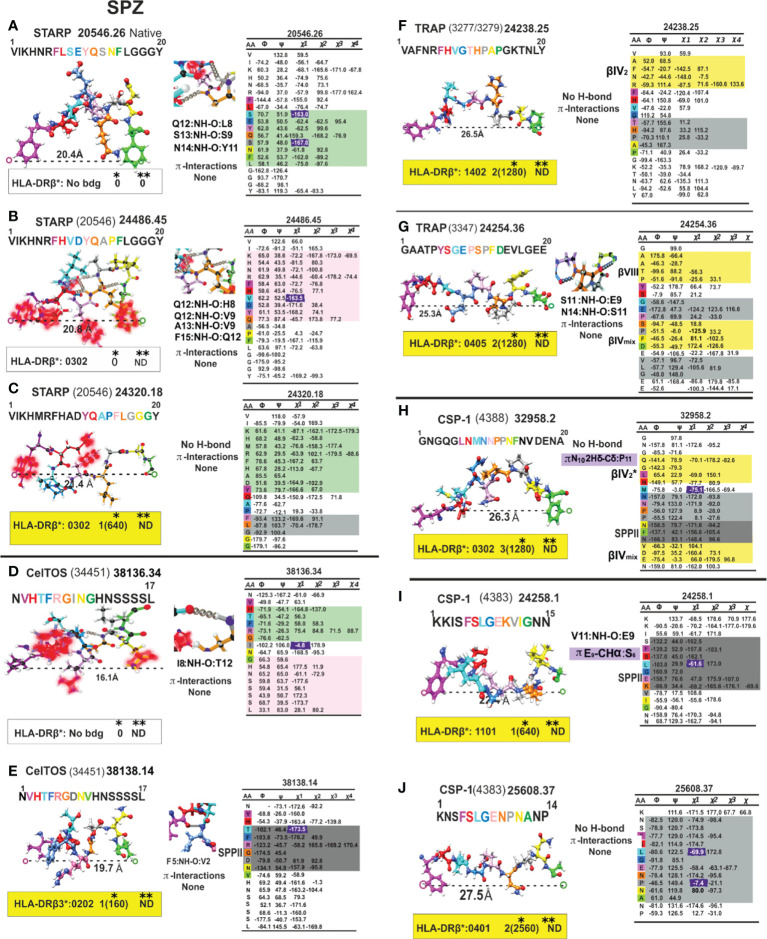
Colour code for peptide binding region, structural and interaction as in **Figure 8**. (*) Nr Monkeys (antibody titers) and (**) Nr of protected monkeys. STARP-, CelTOS-, TRAP- and CSP-1-derived cHABPs and mHABPs. Legend and conventions as in **Figure 8**.

All IMPIPS forming or containing PPII_L_ structures must have a 26.5 Å ± 2.5Å distance between the nine residues to properly fit into HLA-DRβ1* PBR Pockets 1 to 9 (**Figures 5A**, **8**–**12**) (5, 15, 17, 18), according to in-depth structural immunology, immunochemical and immunogenetic studies.

IMPIPS design involved an attempt to avoid β-branched residues (I, V) as PPII_L_ formers for properly fitting into the HLA-DRβ1*PBR since V and I [preferred in β-strands (74)] strongly affect PPII_L_ formation due to bulky β-branched sidechains partly occluding backbone solvation. Interestingly, recent NetMHCIIpan-4.1 predictions have suggested these residues high frequency in their N- and C-terminus Pocket 1 and Pocket 9 in some human MHCII alleles.

Aromatic residues (W, Y, F) (20) were also not preferred in IMPIPS central regions fitting into the HLA-DRβ1*PBR due to their stereo-electron characteristics but accepted in the IMPIPS N-terminal region due to their great relevance in anchoring to the PBR (16).

Negatively-charged residues (D or E, located in the PPII_L_–like turn C-terminus) were not preferred since they induced a more pronounced destabilisation effect on PPII_L_ formation (75) than positively-charged residues at the helix’s N-terminus, both leading to a more compact PPI structure formation (right-handed) (**Figure 7B**). There were ~16.8 Å distances between the 9 residues in the PPI structures which were not appropriate for a proper fit into the HLA-DRβ1* PBR. Both helixes (PPII_L_ and PPI conformation) could be distinguished in peptides by CD spectroscopy (20).

### Molecular orbitals in relevant amino acids

Amino acid molecular orbitals and structures are extremely relevant since they mostly determine molecules’ 3D structures and biological functions.

#### Aromatic and proline residues

CSP-1 4383-derived mHABP 25608.**37** structure was used as a model for explaining the critical behaviour of some aa. The three C6 bonds in Phe 1 (F1) had *sp*2 hybridisation interacting with C1 and C5, each causing a 120° torsion angle, and their 2*px* and 2*py* orbitals promoting a 2*s* orbital electron 2*pz* to form 3*sp*2 hybrid orbitals having the same energy to interact with the 3 neighbouring atoms, one electron thus remaining in the 2*pz* orbital (**Figure 5C**). Hybrid orbital *s* interacted with their analogues from immediately neighbouring C-atoms to form σ-bond (**Figures 5A, C**, yellow orbitals). However, the electron remaining in the *pz* orbital played an important role in F1 resonance structure since the charge became balanced to form the π resonance bond (**Figures 5A, C**, red) resulting from overlapping bonds between the 2*p* orbital located perpendicularly to the inter-nuclear axes. Consequently, as each *p* orbital alternated in such overlapping structure, electron charge was uniformity distributed, therefore inducing π resonance (**Figures 5A, C** blurred red orbitals).

Pro 7 geometry concerns two trigons. The ring horizontally orientated toward the right to contact the TCR CDRβ3 loop formed one trigon involving Cα3, O_C=O_ adjacent to Nα6 and Nα, whilst C_C=O_ was located in the other trigon’s vertex demarked by Cα8. The CC_=O_ plane was not bound to H due to electron delocalisation between O_C=O_, C_C=O_ and Nα2 (since Pro structure is cyclic rather than aliphatic) (**Figures 5A, F, G**). Pro7 confers rigidity on this part of the molecule since Cα and Cσ contiguous to Nα4 are part of this plane. The geometry of each C is tetrahedral.

The above examples (5, 61) show these stereo-electronic patterns’ complexity and their interactions. They clearly demonstrate that minimal subunit-based, chemically-synthesised vaccines are feasible following stereo-electronic principles elegantly discovered and developed by large groups of chemical, physico-chemical scientists and mathematicians.

## Volume and charge

Space, volume and electron charge defined by aa sequence forming HLA-DRβ1*-PBR pockets mostly determine the IMPIPS aa sequence able to fit into the peptide binding groove to induce an appropriate immune response. Dimorphic Pocket 1 having classical Gβ86V dimorphism provides a clear example; the small Gβ86 (90Å^3^) variation enables the fitting of large aromatic residues (F,Y,W order of preference) (**Figure 6A1**), where F is preferred due to electrostatic characteristics.

The Vβ86 variation due to V’s large volume (140Å^3^ vs Gβ86 60Å^3^) occludes this pocket’s lower portion, not permitting aromatic residues whilst allowing only large aliphatic residues (L, I, M and V) (**Figure 6A**). This clearly demonstrates steric impact on immune response induction. The rare variation in *Aotus* Aona DRW1308 allele Aβ86G (**Figure 6A2**) enabled the fitting of large aromatic residues like F and Y, unlike HLA-DRβ1*15 alleles preferring large aliphatic residues (**Figure 6B**, bottom).

The highest HLA-DRβ1* polymorphism occurred in the β-chain, mainly in Pocket 4 where both volume and charge played a critical role determining which aa bind. Qβ70 and the large positively-charged β71R and β74R residues in HLA-DRβ1*03 reduced this pocket’s volume and provided a strongly-positively charged pocket enabling the fitting of small negatively-charged residues like D and sometimes N (**Figure 6A2**). Allele variations Dβ70Q and Qβ74A increased Pocket 4 hydrophobicity enabling the fitting of aliphatic residues like I, L, V, A and some negatively-charged ones like E and D in HLA-DRβ1*0401. However, Dβ70Q and Qβ74A determined that short polar residues like N, S, D were also preferred in Aona DRβW470401, HLA-DRβ1*0401 counterpart (**Figures 6A2, B**).

Increased Pocket 4 volume and electron neutrality was observed in HLA-DRβ1*01 alleles. Qβ70D, Aβ74Q and Vβ78Y determined a preference for large aliphatic residues while Aona DRβ1*W38 preference was for small apolar residues like A, G and some aliphatic residues like L, M and V. Such electron-volumetric difference was more prominent in HLA-DRβ1*15 alleles where Wβ9E, Pβ11F, Rβ13P and small Tβ71, Aβ73 and Aβ74 volume created a large hydrophobic niche. Large, aliphatic (L, M, I) aromatic residues like F and Y were preferred (**Figures 6A2, B**).

Pocket 6 was more orientated toward the α-chain but some β-chain polymorphic residues played critical roles, like Lβ11S and Sβ13 increasing this niche’s space or volume, together with Eα11, Dα60, Qα9, creating a large negatively-charged pocket where large positively-charged residues like K, R, H and Q were preferred in HLA-DRβ1*03 (**Figures 6A3, B**).

The impact of β57D-Rα76 salt bridge was prominent in Pocket 9 regarding β57D**^+^**/β57D**^-^** (V, S, A) polymorphism. Rα76 formed a salt bridge with Dβ57 (in DRβ1*01, 03, 04, 10, 11, 13, 15, 16) 03 (**Figures 6A4, B**); however, when replaced by Vβ57 (in DRβ1*07, 09, 12); Sβ57 (in DRβ1*08,13) or Aβ57 (in DRβ1*14), β57 displacement resulted in a shallow Pocket 9 (wider than deeper), providing Pocket 9 sidechains with greater lateral freedom. If Wβ9 (in DRβ1*01,07,15,16) were the aa in Pocket 9, then large aliphatic residues would be preferred (L, I, V, F), polar ones (K, H, R) preferred with Eβ9 (in DRβ1*03,04,08,10,14) and (V, I) if β9S (in DRβ1*11,13). Such freedom regarding Rα76 would also enable interaction with IMPIPS negatively-charged residues (E,D) like in DRβ1*0405, 0801, 0803 and 1303 (**Figures 6A4, B**).

3D molecular modelling provides an excellent tool for analysing similarities or differences between molecules. This technique was used for modelling *Aotus* (Aona) DRβ molecules comparable to HLA-DRβ1* molecules based on their DNA sequence. Human and *Aotus* space and/or volume are shown for Pockets 4, 6 and 9 (since Pocket 1 dimorphism is shown in **Figure 6A1**) based on X-ray crystallography determined HLA-DRβ1*0401 (PDB code:1J8H), 0301 (PDB code:1A6A), 0101 (PDB code:1DLH) and 1501 (PDB code:1BX2) 3D structure. Stereoelectronic analysis showed strong identity and similarity between HLA-DRβ1* and Aona DRβ alleles where the electrostatic landscape, recognising these pockets’ positively- (blue) and negatively-charged areas (red) and volume, did not change dramatically when the logos for binding motifs to the different alleles (HLA-DRβ1* vs Aona DRβ) were compared (**Figure 6B**). They had tremendous similarity, suggesting that *Aotus* monkey results could be radically extrapolated to humans, involving minimal modification.

## PPII_L_ structure-based IMPIPS design for fitting into the HLA-DRβ1*PBR

### PPII_L_ in IMPIPS

Nine aa fitted perfectly into the PBR to establish 10 to 13 H-bonds (**Figure 5I**) in a fork-like 9-11 atom ring (62) structure (**Figure 5J**) to anchor and stabilise antigen binding to the HLA-DRβ1* PBR; 2 or 3 PPII_L_ would therefore perfectly fulfil such requirements.

nb: since data presented here is based on our peptides’ ^1^H-NMR-determined 3D structure, peptide numbers quoted from here on will indicate where they are located in the figures.

### PPII_L-_related rules for IMPIPS development: Length

The first rule stated that IMPIPS must have a ~26.5 ± 2.5Å distance between the farthest atoms fitting into HLA-DRβ1* PBR Pockets 1 to 9; if such distance were shorter (~22.5 Å ± 1.5), they may bind to another HLA-DR (β3*, β4*, β5*) or HLA-DQ allele family which could induce short-lived protective immunity (76). This might also occur due to p3 and p7 *gauche+* orientation regarding a different presentation platform, like MSP-9-derived 24296 (2150), having very high IFA Ab titres (1:1280) but no protection and a Pockets 1 to 9, 20.6Å distance, having α-helix conformation in the segment fitting into the PBR. Immunogenicity and protective immunity are thus strongly associated with a specific physico-chemical characteristic: length enables specific binding to the HLA-DRβ1*PBR and appropriate TCR-CDRs orientation.

It has not escaped our attention that a large group of native cHABPs (MSP1 1585.44, MSP9 2150.35, SERA5 6737.50, EBA175 1783.9, EBA-175 1815.23 etc) has a highly compact α_R_ structure for performing their biological functions. However, they do not bind to MHC molecules (NetMHCIIpan 4.1), they are too short, are non-Ab, non-protection inducers (i.e. mHABP (MSP-1 13454.40 (1585), EBA-175 24340.25 (1815), CelTOS 38136.34 (34451)) nor α_L_ mHABPs like STARP 24486.45 (20546). They are immunologically silent. Only one mHABP having α_R_ conformation (MSP-2, 24180 (4044)) induced Abs but no protection, where both p3 T and p7N displayed the *gauche^+^* orientation for appropriate TCR interaction and Ab production but their presentation platform was α_R_. The nine residues fitting into the HLA-DRβ1* PBR had a ~16.2Å ± 1.5 Å distance, i.e. too short to fit properly into the PBR (**Figures 8**–**12**).

We have insisted that random coil structures in themselves are neither-Ab nor protection- inducing (MSP-2 4044.13, EBA-175 1758.5, AMA-1 4325.11 cHABPs and mHABP EBA-175 **13786** and 14000.26 (1758) due to their tremendous segmental atomic mobility, suggesting that they have to be further modified to be or contain more stables structures like PPII_L_ helixes to be able to bind to HLA-DR and become immunogenic and protection-inducing. Since random coil structures do not contain H-bonds they are much easier to modify, that being one reasons for our preference.

Most IMPIPS were or contained PPII_L_ enabling them to have an appropriate inter-atom distance (given in Å), i.e. MSP-10014 (1585):27,5 Å; MSP-2 24112 (4044):26.3Å and MSP-2 10008(4044):25.0; MSP-9 24922 (2150):24.5; RESA 9948(6671):24.5; SERA-5 22830 (6725):27.4; AMA-1-20034(4325):23.0; AMA-110022(4313):26.7; EBA-175 13790(1758):29.5 and having p3 and p7 *gauche+* orientation. RESA-13492 (6671):25.2 and AMA-1 22780 (4313):24.0 only had p3 in *gauche+* position and Spz-derived TRAP 24254 (3347):25.3, and 24238 (3277/79):25.6 lacked such orientation. The latter contained a β IV turn, CSP-1 25608 (4383):27.5, inducing very high long-lasting Ab titres against Spz, as determined by IFA 6 months later (**Figures 8**–**12**).

However, some IMPIPS had sPPII conformation and structural distances, like SERA 22834 (6737):24.9; SERA-5 23426 (6754):21.4 and Spz-derived highly immunogenic CSP-1 24258 (4383):22.1; CSP-1 32958 (4388):26.3; and CelTOS 38138 (34451):19.7, all having just p3 *gauche+* orientation. The latter group induced short-lived Ab induction (**Figures 9**, **12**).

This data clearly demonstrates the great relevance of stereo chemical rules (residue length, conformation and gauche+ orientation) for an appropriate immune response against malaria.

## Some critical amino acids’ physico-chemical characteristics in IMPIPS

### Aromatic residues’ physico-chemical characteristics

IMPIPS design demands special care regarding aromatic residues (W, Y, F) due to HLA-DRβ1* PBR Pocket 1 being the main, strongest and deepest pocket (77–79).

The electron cloud associated with aromatic residues (i.e. F) located either side of *pz* orbitals have a uniformly distributed electron density forming a resonant structure, since each *pz* orbital is overlapped by an adjacent *pz* orbital throughout the aromatic ring, thereby forming the negatively-charged, π resonant face (π face) (**Figures 5A, C**).

Concerning Y, both lone-pair electrons from O also orientates one electron pair in the same direction as the ring’s *pz* lobes, increasing its electron density and thereby the π face’s electrostatic effect, being greater in Y than in F and W (61). The Y electrostatic effect is greater than that for Y to H because of the amount of additional atoms in the Y 6-member ring.

Aromatic rings also have abundant interactions (π-π) interacting in a different manner from aliphatic sidechains, such as parallel-displacement, T-shaped, eclipsed face-to-face, stacking, staggering (**Figure 7D**) (49). Such self-association may arise from favourable quadrupole- quadrupole interactions inducing orientation preferences for interaction between two aromatic rings and with other residues, as well as cationic-π interactions, sulphur-aromatic and, recently, anion-π interactions (80). Aromatic rings’ stacking interactions are one of the most common non-covalent interaction motifs in natural proteins and synthetic peptide systems (81).

The propensity of all aa for a W environment shows that small (G, A), negatively-charged (D, E) and polar residues (S, T) avoid the W ring, while long-branched (L, I), i.e. MSP-9 mHABP 24296.16 and 24922.16 (2150) and positively charged ones (R, K), have a strong propensity for W very large aa. Some have a propensity for the π face or the edge of W (Y, F, M and P) (82).

### Proline’s physico-chemical characteristics

Unlike other aa, Pro is linked in a peptide bond as an imino residue where its formation is strongly restricted by a five-membered ring. PPI formation of the backbone is stable only for the shortest (n<3) structures while PPII_L_ prevails in longer polypeptides and neighbouring residues play a predominant role in such structural propensity. A hexaproline peptide’s X-ray crystallography-determined 3D structure (83) revealed that Cγ *exo* had a propensity for n➔π* interaction, whereas Cγ *endo* puckering did not. Cγ *endo* structure forced puckering Pro DOWN at around φ=-75° and ψ=+155 or more, while closest to or below φ=-65° and ψ=+140 highlighted Pro predilection for Cγ *exo* structure puckering Pro UP. AMA-1 4325 (native), 20032 (4325) AMA-1 22780 (4313), 10022 (4313) had p3 Pro puckered UP, as in CSP-1 25608(4383), 32958 (4383) and p7Pro in TRAP 24238 (3277/79), p5Pro in TRAP 24254 (3347) was puckered upwards. All these residues pointing UP (p3, p5, p7) contacted the TCR, suggesting Pro’s critical role in inducing protection against Mrz invasion of RBC or long-lasting Ab production against Spz or SARS-CoV1 infection (73).

## Relevant interactions in IMPIPS

### π-CH interactions

Protein interactions play a significant role in stabilising their 3D structure; for instance, previous studies have demonstrated π-CH interaction (84). Aromatic sidechains are defined by their π faces, Pro-CH bonds are weakly acidic due to their position where hydrogens adjacent to carbonyl (Hα) and amide nitrogen (Hδ) atoms are the most acidic. These hydrogen atoms interact with the π aromatic face and polarised C-H bonds in so-called CH/π interaction (25) establishing *cis* amide bonds (61, 85). This can be clearly observed in STARP-derived 24320.18 (20546) where Y11 dragged Pro14, completely distorting this peptide’s 3D structure, reducing Pockets 1 to 9 distances to 21.4Å and inducing it to bind to HLA-DRβ*0302 alleles leading high, short-lived Ab production (**Figure 12C**).

Aromatic-Pro or Pro-aromatic sequences were thus avoided in our IMPIPS design up to 2 residues upstream or downstream due to their interaction regarding *cis* amide formation.

### Aromatic-aromatic (π-π) interactions

Aromatic self-association could result from the quadrupole-quadrupole interaction giving rise to their orientation preferences (86). Centroid-centroid distances are 4.96 to 5.025 Å when **π-π** interact in edge-face geometry and their inter-planar angles are ~60° (87). They have a 3.4 to 3.6 Å vertical distance and 1.6 to 1.8 Å horizontally when interacting in parallel-displaced orientation (**Figure 7D**); **π-π** can interact in less stable face-to-face orientation (4.96 to 5.025Å) (**Figure 7D**) (88) as in EBA-175-derived 1783 cHABP where Y10 establishes a T-shaped π-π interaction with W14 (**Figure 7D**), having a very short Pockets 1 to 9 distance (17.7Å) (**Figure 10A**). This cHABP is extremely relevant in invasion since it binds to glycan 6 in glycophorin A, mediating RBC invasion.

The four aromatic residues’ electrostatic properties are very different, F-F being less preferred while F-H and F-W are preferred for forming aromatic pairs for parallel conformation with F-H (4.26Å), F-W (4.39Å) and F-Y (5.54Å) distances. Crystallographic analysis of 3D structures has revealed that since hydroxyl groups in Y are an electron-donor group, T-shaped F-Y/Y-F pair conformation is preferred, Y-Y being stronger since both aromatic groups have the electron-donor effect (**Figure 7D**) (88).

### Cation-π interactions

Burley et al. (1986) found a marked tendency for positively-charged amino groups to be preferentially located near W, F, Y aromatic-rings’ π cloud (89), suggesting a self-interaction defined as a cation-π interaction by the 1990s (90).

Singh and Thornton (1990) found extensive 3.6-3.8 Å nitrogen sidechain atom (N, H, K, R) distribution from aromatic residues (91). Karlin et al. (92), confirmed that the guanidinium-aromatic interaction is mostly parallel (stacking), having a predilection for interplanar contacts of the 6-atom ring of W.

W, having nine heteroaromatic-ring atoms, has different contacts along its edge and face and in the π-cationic interaction, where the 6 membered (benzene) ring of indole is preferred over the 5-membered (pyrrole) ring (**Figure 7D**), interacting mainly with NE1 which has the maximum number of contacts. These are mainly hydrogen bonded at the edge, at an average 3.6Å distance (82), as seen in native EBA-175-derived cHABP 1783 between W10 and K14 (**Figure 10A**).

They result from the six C^δ-^ H^δ+^ bond dipoles in aromatic residues producing a region of negative electrostatic potential on the face of the π system causing the geometry to become biased when a cationic sidechain (K or R) is nearby. Burley and Petsko (89) also found that positive residues tend to be positioned within 6Å above the centroid of the aromatic ring (89), as in AMA-1-derived 20034.32 (4325) where F12 pulls R16 (23.0 Å distances between Pockets 1 to 9), making this mHABP a highly immunogenic but short-lived protection-inducer (**Figure 11C**). Something similar happened with CelTOS-derived 38138 (34451) where F4 dragged R5, inducing an sPPII structure, having a short distance between Pockets 1 to 9 binding to HLA-DRβ3*0202 and poor short Ab induction. N and Q only make polar-π interactions, as in STARP-derived 24486 (20546) (**Figure 12B**), while R and K participated in much stronger cation-π interactions.

### NH···N hydrogen bonds inducing H imidazole nitrogen

H imidazole nitrogen (Nδ/Nε) participate as hydrogen bond acceptor or donor in protein interactions. H is one of the three basic residues (H, R, K) that can form salt-bridges with acid residues (D, E), i.e. AMA-1 (13766.29 (4313)), and single N-H ···Nδ/Nε hydrogen bond with the main chain N-H groups of *i+2* or bifurcated, if *i+3* induced by Pro is preferred in 40% of the examples if it is in *i+1* position (93).

### Sulphur-containing residue (M) stereochemistry

Sulphur atoms in sulphur-containing aa (C and M) sometimes behave as electrophile and other times as nucleophile. Protein structure study has revealed that M is close to peptide backbone carbonyl (even carboxylate) oxygen atoms, suggesting direct contact with the oxygen (acting as a nucleophile) approaching sulphur (electrophile). The sulphur atom is located above the oxygen atom, having relatively well-defined orientation. Native MSP-2-derived cHABP 4044.13 provides a clear example of π-S interaction, having 18.3 Å in Pockets 1 to 9 distance (**Figure 8E**). Such stereochemistry becomes very different when divalent sulphur interacts with an aromatic sidechain. The sulphur atom becomes orientated towards the π face if located on the π-electron-rich face, particularly towards Y ring t N atoms (94), also observed in cHABP 4044.13 (**Figure 8E**).

M and C are usually close to aromatic residues, suggested as being protein stabilisation structures (95), several geometries usually being observed.

## IMPIPS structural and stereo-electronic principles

### Aromatic-aromatic interactions in IMPIPS

A previous aromatic residue in AMA-1 4313-derived 13766.29, 13480.50, 22780.1 and 10022.43 -p1 (W, W, Y, Y) orientated p1F downwards in a *trans* conformation (**Figures 11E–H**), clearly demonstrating π-π interactions with aromatic in Pocket 1. Only 22780.1 and 10022.43 preceded by Y in p-1 were highly immunogenic and protection-inducers; the others, preceded by W in *i-1*, induced high Ab titres but **no** protection due to H-bond formation with pF1. The strength of these interactions followed a W>H>Y>F pattern, the first two being strong enough to make significant contributions to protein structure (96).

Interestingly, AMA-1 4313-derived 10022.43(**Figure 11H**) IMPIPS had a IV3 β turn preceding the PPII_L_ structure in p-1Y. However, such structure completely disappeared in highly immunogenic, non-protection-inducing 13766.29 analogue (**Figure 11E**) having –p1W; Pocket 1F, p2D motif being recovered as a type I β-turn, also in analogue 13480.50 (**Figure 11F**) having high Ab production but no protection induction where p-1W was present but the PPII_L_ structure contained p2L.

Analysing distances between φ angles in residues participating in π/CH or π-π interactions indicated that cut-off distances greater than 5Å (between 2 aromatic rings centroids) were more prevalent in π/CH, having T-shaped orientation, while stacked orientation in π-π interactions were more prevalent at smaller cut-off distances like occurred in a previous research (49).

This situation was quite similar but more pronounced regarding mHABP 13766.29 which was also highly immunogenic but non-protection-inducing, where D11 C=O negatively-charged p2 resonant structure strongly attracted two weakly positive-charged Pro 12/Hδ atoms (**Figure 11E** and zoom), modifying *gauche+*p3P orientation.

Such π/CH interactions did not occur in highly immunogenic but non-protection-inducing mHABP 13480.50, where p2 was replaced by aliphatic L2 which does not establish any interactions with Pocket 1F or p3P (**Figure 11F** and zoom). A difference between these 3 mHABPs having almost the same distances between the farthest atoms fitting into HLA- DRβ1*PBR Pockets 1 to 9 (26.7, 26.3, 27.7 Å) arose from the presence/absence of this type of CH/π interaction, and impact on p3, p7 residue orientation to interact with the TCR. This confirmed the relevance of such interactions regarding protein/peptide structure and immunological function.

Supporting such data, MSP1 1585-derived 11860.25 and 10014.35 (highly immunogenic and protection-inducing IMPIPS) having the p1Y, p2H motif (10014.35 shown in **Figure 8D** and zoom) had π/CH interactions between the p2H partially resonant tiara-like structure and Pro4 Hδ and Hα atoms (**Figure 8D**). This situation was modified in 11860.25 (**Figure 8C** and zoom) by p3M *versus* p3V in 10014.25 (**Figure 8D** and zoom), CH/π being the interacting force with p4P. This was stronger than sulphur-containing p3M folding the molecule differently, making it shorter (22.6Å) and inducing strong but short-lived, memory-related, protection-inducing immunity (97).

A π-sulphur interaction between p1Y and p3M was induced in MSP-2 4044-derived 24112.39 IMPIPS (**Figure 8H**), bringing both residues close and establishing an interaction between p1Y centroid and p3M sulphur. A ~5Å distance pulled this p3 TCR-contacting residue down, suggesting that 24112.39-induced protective immunity was strongly mediated by appropriate p5I, p7R and p8S TCR orientation, even though p3M and p7R had gauche+ orientation.

Spz STARP 20546-derived 24320.18 (**Figure 12C**) provided a further example of π/CH interaction, having two HLA-DR binding registers. One of them bound more strongly to HLA-DRβ3*0201/0302 than HLA- DRβ1* 0302, the latter inducing very high, long-lasting Ab titres against Spz (IFA) and recombinant protein STARP (WB). This mHABP had a 21.4 Å distance between Pockets 1 to 9, having a π/CH interaction between p1Y centroid and both p4P Hα atoms orientating both rings in *cis* conformation. The attraction exerted by p1Y electron-rich π face was stronger than the electron-poor p5F π face, confirming that shown by others, making this Spz mHABP highly immunogenic, but a short-lived immunity-inducer.

### TCR-contacting residues’ stereo-electron characteristics

Our previous studies involving 20 very high, long-lasting, antibody-inducing IMPIPS have shown that residues in p3 and p7 must have *gauche^+^* sidechain orientation for MHCII-p-TCR complex formation and that all residues in p3 must have aliphatic or apolar characteristics (23). Such orientation is different in all non-protection-inducing mHABPs which are just immunogenic but whose p3 sidechains have gauche^-^ orientation, or have both of them in an inappropriate platform. Examples would be MSP2 4044-derived 24180.41, having both p3I and p7N in *gauche^+^* orientation but in α-helix conformation (**Figure 8F**), or those in SERA-derived 14096.12 (6737) in random coil conformation (**Figure 9G**) or having only one in appropriate orientation, as in EBA-175-derived 14000.26 (1758) having random coil conformation or only one properly placed in a PPII_L_-like structure, 14004.22 (1758) (**Figure 10I**). This suggested that both MHCII-p-TCR interactions needed to be perfectly orientated to induce a protection-inducing immune response, different orientation thus allowing protectivity and immunogenicity to be clearly differentiated.

Structural studies with ~50 MHCII-p-TCR complexes have shown remarkable TCR diagonal topology concerning the p-MHCII-PBR complex, having ~100° variability. CDR1α (white, **Figure 3G**) contacted a peptide’s N-terminus, CDR1β (light green, **Figure 3G**) a peptide’s C-terminus, CDR2α (orange in **Figure 3G**) and CDR2β (dark green) contacted conserved aa in the MHCII (98–101). Their interaction was modulated by CDR3α (yellow) and CDR3β (red) loops (i.e. CDR3 editing where immune activation does not occur if upwardly-pointing peptides are not properly orientated (102). Such geometric constrains regarding MHCII- p-TCR docking footprints compatible with signalling suggested that TCR signalling can be modulated by the complex architecture of MHC II-peptide orientation.

## Some other antimalarial vaccines

One hundred and twenty-three biologically-derived antimalarial vaccines targeting Spz, liver stage, pre-erythrocyte vaccines or blood stage and transmission blocking vaccines have been tested in clinical trials, some on large human populations. Such biological vaccines use whole, genetically-attenuated, or radiation attenuated (103, 104), vector-based Spz or Mrz (105, 106), recombinant proteins (107, 108) etc. Thirty more antimalarial vaccine candidates involving the same methodologies are now in clinical trials, but none are chemically-synthesised. Those having completed trials have been shown to provide negative or very poor protective vaccine efficacy, as elegantly reviewed in (109, 110).

The most recent example of such failure was the highly-publicised and very expensive RTS-S AS01 (~2 billon dollars invested) (111), a hybrid vaccine tested on thousands of African children that provided low protective efficacy (29% to 0%), protection being understood as the presence of < 5,000 parasites per microliter (1 parasite x 1,000 RBC). RTS-S ASO1 did not protect children <1 year of age (112). Recent 5-year follow-up analysis revealed that RTS S-ASO1 had -43.0% to -56.0% protective efficacy (113). The WHO launched this recombinant vaccine on October 6^th^ 2021 after a large-scale, lengthy trial involving ~600,000 children in 3 African countries; it concluded that RTS-S AS01 provided ~32% protection in children after 4 doses delivered throughout one year. This is a very poor result compared to the first chemically-synthesised antimalarial vaccine (SPf66) that provided ~35% protective efficacy 25 years ago in largescale human trials performed in different countries involving different ethnic groups, being really the first anti-*P. falciparum* malaria vaccine (3, 114).

The forgoing clearly suggests that vaccine development is a more elaborate issue than just vaccinating people with any biological product, therefore requiring in depth physical, chemical, immunological and mathematical analysis, a task we have undertaken for human and animal welfare.

## Conclusions

### The alphabet of synthetic vaccine development

This review has shown the feasibility of chemically-synthesised, anti-malarial vaccines, following previously demonstrated stereo-electronic principles (3, 5, 11, 17) and those shown here, developed over the last 40 years:

- Short (~20 mer-long) chemically-synthesised peptides from conserved, biologically-relevant proteins having high host cell binding activity (cHABPs) must be identified as immune response targets;
- However, as cHABPs are immunologically silent, they must be specifically modified (mHABPs) to make them immunogenic and protection inducers;
- mHABPs must be specifically modified to fit into human (HLA-DRβ1*) and *Aotus* (Aona DR) peptide binding regions (PBR);
- Mathematical platforms nowadays can predict with relative accuracy which binding motifs mHABPs must have to bind MHCII molecules;
- mHABP modifications must fulfil specific, previously-demonstrated, stereoelectronic principles and rules;
- mHABPs must have a 26.5 ± 2.5Å inter-atom distance between aa fitting into Pockets 1-9 of MHCII molecules’ PBR;
- Residues fitting into PBR Pocket 1 must be aromatic (F, Y, W order of frequency) or large aliphatic ones (L,I,V) due to this highly hydrophobic pocket’s genetic dimorphism (Gβ86V);
- Regarding Pocket 4 and 6 strong polymorphism, aa must be selected according to their charge, volume and orientation to properly fit into these pockets (i.e. a fork-like 11-atom ring or 6-atom trigon is established together with Pockets 1 and Pocket 9 aa side chains to anchor and stabilise a peptide in the PBR);
- Specific aa stereoelectronic requirements must be fulfilled regarding Pocket 9 due to β57D**^+^**/β57D**^-^** (V, S, T, A) polymorphism for a proper fit into this pocket, i.e. different sidechain orientation for a perfect fit;
- TCR-contacting residues must also have specific stereochemical characteristics to form the MHCII-p-TCR trimolecular complex: i.e. p2 residues must be polar, preferentially negatively-charged, p3 must be apolar, aliphatic or Pro puckered UP having *gauche^+^* orientation, p5 must be charged, p7 aliphatic (having a strong preference for Pro also puckered UP and *gauche^+^* orientated) and p8 could be aliphatic, polar or negatively-charged;
- mHABPs must have or contain PPII_L_ structure to properly fit into the MHCII-PBR;
- PPII_L_ propensity must involve P>>L>R>A>K>M/D>Q/H,E>G/N/S/T/V/I/F/Y/W aa preference;
- β-branched and small polar residues tending to establish (SC-BB) interactions must be avoided in the PBR due to their tendency to establish H-bonds among themselves, thereby disturbing PPII_L_ structure formation; and
- Special IMPIPS characteristics must be taken into account when designing them as relevant aa are involved in immune activation like Pro while others have PPII_L_ formation disruption capability, i.e. those establishing π-CH, π-cation, π-sulphur, π-π and n➔π interactions.

The forgoing data strongly supports a logical and rational methodology regarding the concept of minimal subunit, multiepitope, multistage chemically-synthesised vaccine development.

## Author contributions

MEP Conceptualization, data analyses and writing the manuscript. AB, MA, and MAP analysed and interpreted the raw data and helped draft. MEP, AB, MA, MAP, CS, JA-C, AM-V, and MV: All authors have read and approved the final manuscript.

## Acknowledgments

The authors wish to thank the Universidad de Ciencias Aplicadas y Ambientales (U.D.C.A) for funding.

## Conflict of interest

The authors declare that the research was conducted in the absence of any commercial or financial relationships that could be construed as a potential conflict of interest.

## Publisher’s note

All claims expressed in this article are solely those of the authors and do not necessarily represent those of their affiliated organizations, or those of the publisher, the editors and the reviewers. Any product that may be evaluated in this article, or claim that may be made by its manufacturer, is not guaranteed or endorsed by the publisher.
